# Revascularization Strategy in Myocardial Infarction with Multivessel Disease

**DOI:** 10.3390/jcm13071918

**Published:** 2024-03-26

**Authors:** Alexander Jobs, Steffen Desch, Anne Freund, Hans-Josef Feistritzer, Holger Thiele

**Affiliations:** Department of Internal Medicine/Cardiology and Leipzig Heart Institute, Heart Center Leipzig at University of Leipzig, Strümpellstr. 39, Russenstraße 69a, 04289 Leipzig, Germany

**Keywords:** acute coronary syndrome, STEMI, NSTEMI, revascularization, multivessel disease

## Abstract

The proportion of patients with multivessel coronary artery disease in individuals experiencing acute coronary syndrome (ACS) varies based on age and ACS subtype. In patients with ST-segment elevation myocardial infarction (STEMI) without cardiogenic shock, the prognostic benefit of complete revascularization has been demonstrated by several randomized trials and meta-analyses, leading to a strong guideline recommendation. However, similar data are lacking for ACS without ST-segment elevation (NSTE-ACS). Non-randomized data suggesting a benefit from complete revascularization in non-ST-segment elevation myocardial infarction (NSTEMI) are prone to selection bias and should be interpreted with caution. A series of large randomized controlled trials have been initiated recently to address these open questions.

## 1. Introduction

Acute coronary syndrome (ACS) encompasses a broad clinical spectrum, spanning from patients with unstable angina to those experiencing a myocardial infarction, as well as individuals with infarct-related cardiogenic shock and cardiac arrest [1]. Typically, a stenosis in a coronary artery is causative of acute ischemia. Such stenosis can develop due to progressive atherosclerotic plaque formation or may occur acutely due to complete or partially occlusive thrombus formation caused by plaque erosion or rupture. If myocardial necrosis occurs within a vascular territory due to ischemia following a stenosis or occlusion, this is termed a myocardial infarction. The causative pathological mechanism often differs between patients with ST-segment elevation myocardial infarction (STEMI) and non-ST-segment elevation myocardial infarction (NSTEMI) [2,3]. Acute complete occlusion of a coronary artery due to plaque rupture without significant collateral blood supply to the downstream vascular region typically results in a STEMI, leading to the development of a transmural infarction. If there is residual coronary blood flow due to a partial occlusion caused by plaque erosion or sufficient collateral circulation, this usually results in an NSTEMI with the development of a subendocardial but also sometimes transmural infarction. The coronary lesion responsible for this is termed the “culprit lesion.” When coronary arteries or their major branches beyond the culprit lesion are more than 50% stenosed, it indicates by consensus multivessel disease. Depending on the number of affected supply areas, coronary artery disease is classified more specifically as two-vessel or three-vessel disease. The prevalence of coronary multivessel disease varies based on the ACS type, as well as according to the patient’s risk profile and age [4,5,6].

Patients with STEMI are generally younger and have fewer comorbidities compared to patients with NSTEMI. Typical comorbidities include obesity, type 2 diabetes, and chronic kidney disease. The higher age and more frequent comorbidities in NSTEMI patients are accompanied by a higher prevalence of multivessel disease in this subgroup of ACS. For patients with ACS without ST-segment elevation (NSTE-ACS), which includes unstable angina and NSTEMI, the prevalence of multivessel disease ranges up to 70%, depending on the examined cohort [4,6]. In STEMI patients, it is around 50% [5]. In the case of infarct-related cardiogenic shock, the prevalence is highest, at approximately 80% [7].

Revascularization by percutaneous coronary intervention (PCI) is the standard of care for the culprit lesion in STEMI as well as NSTEMI, irrespective of the hemodynamic situation. However, the revascularization strategy for non-culprit lesions in multivessel disease remains a matter of debate.

With regard to the optimal revascularization strategy, the following questions arise after treating the culprit lesion concerning the non-culprit lesions:

(1) Should relevant non-culprit lesions be revascularized in addition to the culprit lesion (basic question)?
(2) What is the optimal timing for complete revascularization: either immediate complete revascularization or staged complete revascularization?
(3) If staged revascularization is performed, should it be performed either during the index hospital stay or at some interval within a defined time window as part of elective readmission?
(4) How should complete revascularization be guided: angiographically, based on physiological parameters indicating hemodynamic relevance (e.g., FFR, RFR, …), or based on morphological characteristics identifying vulnerable plaques (e.g., OCT)?
(5) Are there differences in these strategies between patients with STEMI, NSTEMI, and those with or without cardiogenic shock?

The following sections present and discuss the available scientific evidence on these questions.

## 2. Multivessel PCI in STEMI

**Figure 1:**
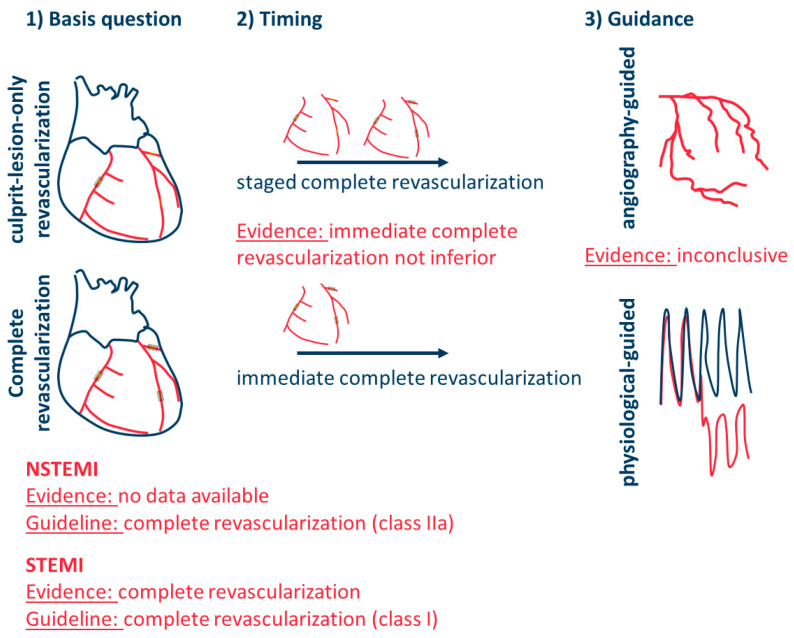
Overview of the three major questions regarding the optimal revascularization strategy in myocardial infarction with multivessel disease. (1) The main question of whether complete revascularization should be pursued is convincingly clarified for STEMI, especially by the results of the COMPLETE trial. In the case of NSTEMI, there is currently no scientific evidence on this issue. (2) Regarding the timing of complete revascularization, the data suggest that immediate complete revascularization is not inferior to staged complete revascularization. The arrows pointing to the right represent the temporal sequence. In a staged complete revascularization, at least two revascularization procedures are performed, while in immediate complete revascularization, all relevant stenosed vessels are treated in one procedure. (3) The data on guidance for complete revascularization are complex and inconclusive. NSTEMI = non-ST-segment elevation myocardial infarction; STEMI = ST-segment elevation myocardial infarction.

In hemodynamically stable STEMI patients with multivessel disease, complete revascularization is recommended, either during the index procedure or as a staged procedure within 45 days (Figure 1) [1]. This upgrade in the recommendation level in current guidelines is based mainly on the COMPLETE trial, which not only demonstrated a decrease in repeat revascularizations but also a reduction in the reinfarction rate [8]. Newly added to the European Society of Cardiology (ESC) guidelines is the recommendation that PCI of the remaining stenoses should be guided by angiographic severity. This is primarily attributed to the FLOWER-MI study, which showed no advantage of fractional flow reserve (FFR)-guided PCI over purely angiography-guided PCI for non-culprit lesions in STEMI patients with multivessel disease [9]. In a subanalysis of this trial, non-treatment of angiographically relevant stenoses with a negative FFR measurement (>0.80) was associated with a higher event rate [10]. This could be because FFR might be a false negative in the setting of acute MI, as described in a substudy of the REDUCE-MVI trial by van der Hoeven et al. [11]. The authors determined the FFR of non-culprit lesions immediately after PCI of the culprit lesion and again after one month. It was found that in the acute phase, 15% of stenoses were hemodynamically relevant compared to 26% of the stenoses at follow-up. Interestingly, the blunted acute hyperemic response correlated with the infarct size derived from cardiac magnetic resonance imaging. In line with the increased event rate in FFR-related deferral of angiographically relevant stenoses in the aforementioned subanalysis, the optical coherence tomography (OCT) substudy of the COMPLETE trial showed angiographically relevant lesions more commonly exhibiting vulnerable plaque morphology like a thin-cap fibroatheroma than non-obstructive lesions [12]. In DANAMI-3-PRIMULTI, FFR-guided complete revascularization was compared with culprit lesion-only revascularization in hemodynamically stable STEMI patients. In FFR-guided complete revascularization, 31% of angiographically relevant stenoses (i.e., stenosis > 50%) were deferred due to FFR >0.8 [13]. In the FORZA trial, 350 patients with at least one angiographically intermediate coronary lesion were randomized to either FFR- or OCT-guided PCI [14]. The primary major adverse cardiac events or significant angina at 13 months of follow-up were significantly less frequent in the OCT-guided group compared to the FFR-guided group (8.0% vs. 14.8%, respectively; *p* = 0.048). Even though only about 19% of these patients had an ACS, this emphasizes the importance of plaque morphology beyond acute functional relevance for avoiding future cardiovascular events. The angiographic significance could be a surrogate for vulnerable plaque morphology, suggesting that angiography-guided PCI with consequent plaque sealing might be superior to FFR-guided PCI of non-culprit lesions.

The Korean FRAME-AMI trial also compared FFR-guided PCI with purely angiography-guided PCI of non-culprit lesions in patients with an acute MI. A total of 562 patients were randomized 1:1, of whom 47% had a STEMI and 53% had an NSTEMI. Unlike FLOWER-MI, the risk for the primary endpoint of death, reinfarction, and repeat revascularization after a median follow-up of 3.5 years was lower in the FFR-guided PCI group than in the angiography-guided PCI group (7.4% vs. 19.7%; hazard ratio, 0.43; 95% confidence interval, 0.25–0.75; *p* = 0.003) [15]. However, the results of this trial should be interpreted with caution due to premature study termination as a result of slow recruitment, with only 562 of the initially planned 1292 patients randomized. A substudy further revealed that the treatment effect on the primary endpoint was not modified by the severity of the stenoses, determined by quantitative coronary angiography (QCA) [16].

## 3. Theoretical Harms and Benefits of Culprit Lesion-Only and Complete Revascularization

A culprit lesion-only strategy, compared to a complete revascularization strategy, would imply fewer interventions for patients, with cumulatively less contrast medium and fewer implanted stents. This could potentially lead to fewer access site complications (e.g., bleeding events), contrast-associated acute kidney injury, and stent thromboses. However, these events themselves are very rare, and in the COMPLETE trial, there was no signal for an accumulation of such events [8]. Specifically, contrast-associated acute kidney injury was not more frequent in the complete revascularization group compared to the culprit lesion-only group (1.5% vs. 0.9%). Furthermore, the rate of stent thrombosis was not significantly different between the groups (1.3% vs. 0.9%) [8]. Conversely, there is a benefit in preventing future MIs through plaque sealing within the complete revascularization group, as observed in the COMPLETE trial. However, NSTEMI is a disease entity clearly distinct from STEMI, as described in the previous section. Therefore, results from STEMI trials cannot be extrapolated one by one to the NSTEMI cohort. For instance, immediate complete revascularization compared to culprit lesion-only revascularization with possible staged complete revascularization has proven to be even inferior in cardiogenic shock in the CULPRIT-SHOCK trial [7]. Additional PCI of non-culprit lesions was performed in close to 1/3 of the 50% survivors in the initial culprit lesion-only group. In contrast, non-culprit lesion PCI was only permitted if pre-defined bailout criteria were met in the COMPLETE trial. This resulted in a crossover rate of 4.7% within the initial 45 days and 19.3% throughout the entire duration of the trial in the culprit lesion-only group [8,17]. A higher susceptibility to adverse outcomes from immediate complete non-culprit lesion revascularization in NSTEMI, akin to cardiogenic shock, may be possible and could be attributed to a greater burden of comorbidities and older patient age. NSTEMI patients might face increased vulnerability to complications like access site issues or contrast-associated acute kidney injury, potentially offsetting any benefits from plaque sealing.

## 4. Multivessel PCI in NSTEMI

NSTE-ACS is the most common form of ACS, accounting for approximately 70% of all cases [18]. Despite the widespread use of PCI, which has been associated with a significant decrease in mortality, the mortality of these patients remains substantial [19]. In Germany, in the year 2019, 4.7% of all deaths were attributed to an acute MI.

According to the recently revised ESC guidelines for ACS, released in August 2023, complete revascularization should be considered in patients with NSTE-ACS and multivessel disease [1]. However, this recommendation is based solely on observational studies and meta-analyses of non-randomized studies, and therefore, it is a recommendation with an evidence level of C (Figure 1).

Unlike STEMI, currently, no randomized data exist comparing complete revascularization with culprit lesion-only revascularization exclusively in patients with NSTE-ACS or NSTEMI.

As already stressed above, not only do the clinical characteristics of patients with STEMI differ from those with NSTEMI, but often, also the plaque morphology. For example, intravascular OCT has shown increased plaque vulnerability (i.e., more plaque ruptures) in STEMI patients compared to NSTEMI patients [3]. Therefore, the results of STEMI patients cannot be extrapolated to NSTEMI patients, even though observational studies also indicate a benefit with complete revascularization in NSTEMI patients. However, these positive signals in favor of complete revascularization could be due to selection bias. It is well-known that observational and randomized data may lead to different conclusions [20].

A meta-analysis of 15 non-randomized studies found a lower rate of cardiovascular events and deaths with simultaneous complete revascularization compared to revascularization of the culprit lesion alone [21]. A national study involving 105,866 patients with NSTE-ACS and multivessel disease undergoing PCI in the United States indicated that complete single-stage revascularization appears to be associated with similar in-hospital outcomes as culprit vessel-only PCI [4]. According to data from the British Cardiac Intervention Society PCI database, among 21,857 patients with NSTEMI and multivessel disease, single-stage complete revascularization demonstrated a survival benefit when compared to culprit-only PCI [6].

Recently, the so-called FIRE trial has been published [22]. This trial showed the benefit of physiologically-guided complete revascularization of non-culprit lesions over culprit lesion PCI alone in patients ≥75 years of age. However, this is a mixed cohort of older NSTEMI and STEMI patients (approximately 65% and 35% of patients, respectively), making it challenging to extrapolate the study results to the NSTEMI population in clinical practice. The FRAME-AMI trial observed a similar result in younger patients with MI (approximately 47% with STEMI and 53% with NSTEMI) [15]. However, as discussed above in more detail, these results are in contrast to the FLOWER-MI trial, which randomized only STEMI patients [9].

To date, no randomized trial has exclusively examined the optimal revascularization strategy in NSTEMI patients with multivessel disease. Only trials designed to investigate the optimal timing of complete revascularization, rather than addressing the basic question of whether or not to treat non-culprit lesions, were conducted in NSTEMI patients. These trials will be discussed in the next chapter.

## 5. Timing of Complete Revascularization

In a subgroup analysis of the COMPLETE trial, the intended timing of non-culprit lesion PCI, whether conducted staged during the index hospitalization or after hospital discharge, did not alter the impact of complete revascularization compared to culprit lesion-only revascularization [8]. A more detailed post-hoc analysis of the timings concluded that percutaneous revascularization of non-culprit lesions, whether performed early during the index hospitalization or several weeks after discharge, is linked to similar reductions in the composite outcome of reinfarction or cardiovascular death when compared to culprit lesion-only PCI [23]. This suggests that the benefits of non-culprit lesion PCI become apparent over a more extended follow-up period through the prevention of reinfarctions via sealing vulnerable plaques.

**Figure 2:**
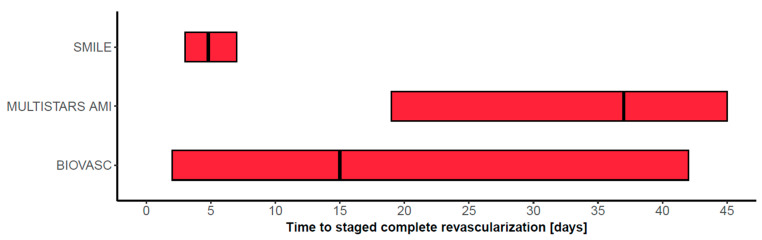
The time frame defined in each trial protocol for complete revascularization is depicted as a red bar. The black line within the bar represents the actual median timing.

Three RCTs (SMILE, BIOVASC, MULTISTARS) specifically examined the timing of complete revascularization. The intervention group in all studies underwent immediate complete revascularization, and the control group underwent staged complete revascularization. The timeframe for these interventions varied among the studies (Figure 2). Complete revascularization was primarily angiography-guided. Beyond the timing of staged complete revascularization, these studies significantly differ in terms of the composition of the infarct entity of the included patients. While the SMILE trial only enrolled patients with NSTEMI, and MULTISTARS included only patients with STEMI, the BIOVASC cohort was heterogeneous (approximately 40% STEMI, 52% NSTEMI, and 8% unstable angina).

The SMILE trial suggested the benefit of single-stage complete revascularization over a multi-stage approach concerning the combined endpoint of major adverse cardiovascular and cerebrovascular events [24]. This advantage was primarily attributed to a lower rate of repeat revascularizations in the single-stage revascularization group. However, it is important to note that the trial was too small to draw a definitive conclusion.

Both BIOVASC and MULTISTARS concluded that immediate complete revascularization is non-inferior to staged complete revascularization based on their respective primary endpoints [25,26] (Table 1 for details). A closer look at the supplement of the MULTISTARS AMI trial reveals that the only type of MI with a significant difference between groups is the MI associated with PCI (type 4a). Its occurrence was 12 times more frequent in the staged complete revascularization group than in the immediate complete revascularization group, and none of these events was detected as a result of the index procedure. MI type 4a was defined according to the fourth universal definition of MI [27]. Due to the definitions, detecting a type 4 MI in the acute MI setting is very challenging and possibly hardly distinguishable from the primary type 1 MI, so the increased number of type 4a MIs in the staged complete revascularization group could be the result of a detection bias.

Also, the endpoint of unplanned ischemia-driven revascularization was more frequent in patients with staged complete revascularization than in those with immediate complete revascularization. However, the cumulative incidence function curves for this endpoint diverged within the first 45 days and then ran almost parallel. Consistent with this, a landmark analysis of the primary endpoint showed that the event rate difference between the groups widened within the first 45 days but subsequently showed no significant difference in event rates. This is not surprising, considering that the median time from randomization to the staged intervention was 37 days. The pattern was similar for BIOVASC. Here, 1 type 4a MI was detected in the immediate complete revascularization group, but 11 were detected in the staged complete revascularization group. Type 4a MIs accounted for a total of 32% of all non-fatal MIs in the staged complete revascularization group. Correspondingly, both the cumulative incidence function curves for the primary endpoint and for non-fatal MI diverged within the first 30 days and then ran almost parallel or even converged again. A similar challenge is known in randomized controlled trials (RCTs) examining the optimal timing of invasive coronary angiography in NSTE-ACS [28]. Therefore, the interpretation of procedure-related MIs and repeated revascularizations is difficult for studies comparing the optimal timing of revascularization, especially when one group undergoes revascularization at the time of the index acute MI. In conjunction with the COMPLETE trial data discussed earlier, it can be concluded that immediate complete revascularization is likely not inferior concerning the endpoints investigated. However, it probably does not confer an advantage on hard clinical endpoints, and a well-planned complete revascularization can be undertaken staged without the need for an immediate complete revascularization during on-call hours.

**Table 1:** Summary of trials studying the revascularization strategy in patients with myocardial infarction and multivessel coronary artery disease. ^1^ stenosis > x% indicates that the degree of stenosis is angiographically > x% based on visual estimation; Abbreviations: cFFR = contrast fractional flow reserve; CompR = complete revascularization; COR = culprit lesion-only revascularization; CV = cardiovascular; FFR = fractional flow reserve; FU = follow-up; HHF = hospitalization for heart failure; IDR = ischemia-driven revascularization; iwFR = instantaneous wave-free ratio; MI = myocardial infarction; NSTEMI = non-ST-segment elevation myocardial infarction; RR = repeat revascularization (with study specific definitions); STEMI = ST-segment elevation myocardial infarction; QFR = quantitative flow ratio.

Trial	Year	n	Population	Comparison	Indication for Complete Revascularization ^1^	Primary Endpoint and Result
Complete revascularization vs. culprit lesion-only revascularization						
PRAMI [29]	2013	465	STEMI	COR vs. immediate CompR	stenosis > 50%	CV death, MI, refractory angina within a mean FU of 23 months HR 0.35; 95% CI 0.21–0.58 *p* < 0.001
CvLPRIT [30]	2014	296	STEMI	COR vs. immediate/staged CompR	stenosis > 70% monoplan or > 50% biplan	death, MI, HHF, IDR @1 year HR 0.45; 95% CI 0.24–0.84 *p* = 0.009
DANAMI-3-PRIMULTI [13]	2015	627	STEMI	COR vs. staged (index hospitalization) CompR	FFR ≤ 0.80 or stenosis > 90%	death, MI, IDR within a median FU of 287 months HR 0.56, 95% CI 0.38–0.83 *p* = 0·004
Compare-Acute [31]	2017	885	STEMI	COR vs. immediate CompR	FFR ≤ 0.80	death, MI, revascularization, cerebrovascular events @1year HR 0.35; 95% CI 0.22–0.55 *p* < 0.001
COMPLETE [32]	2019	4041	STEMI	COR vs. staged (index hospitalization or with 45 days) CompR	stenosis > 70% or FFR ≤ 0.80 in stenosis 50–70%	CV death or MI within a median FU of 3 years HR 0.74, 95% CI 0.60–0.91 *p* = 0.004
FIRE [22]	2023	1445	Age ≥ 75, STEMI (35%), NSTEMI (65%)	COR vs. single-stage or multi-stage physiology-guided CompR	FFR ≤ 0.80 or iwFR ≤ 0.89 or cFFR ≤ 0.85 or QFR ≤ 0.80	death, MI, stroke, or IDR @1y HR 0.73, 95% 0.57–0.93 *p* = 0.01
COMPLETE-NSTEMI	recruiting	3390	NSTEMI	COR vs. single-stage or multi-stage CompR	stenosis > 70% monoplan or > 50% biplan	CV death or MI within the FU period of min. 1 to max. 3 years
Timing of complete revascularization						
SMILE [24]	2016	584	NSTEMI	single-stage vs. multi-stage (index hospitalization) CompR	Angio, FFR optional	death, MI, rehospitalization for unstable angina, RR, or stroke @1y HR 0.55, 95% CI 0.36–0.83 *p* = 0.004
BIOVASC [26]	2023	1525	STEMI (40%), NSTEMI (52%), UA (8%)	single-stage vs. multi-stage (index hospitalization or within 6 weeks) CompR	stenosis > 70% or FFR ≤ 0.80	death, MI, IDR, or cerebrovascular events @1y HR 0.78, 95% CI 0.55–1.11 *p* = 0.0011 for non-inferiority
MULTISTARS [25]	2023	840	STEMI	single-stage vs. multi-stage (within 19 to 45 days) CompR	stenosis ≥ 70% FFR optional	death, MI, stroke, IDR, or HHF @1y HR 0.52, 95% CI 0.38–0.72 *p* < 0.001
Guidance of complete revascularization						
FLOWER-MI [9]	2021	1171	STEMI	FFR-guided CompR vs. angio-guided CompR	FFR ≤ 0.80) vs. stenosis ≥ 50% (staged ~96%)	death, MI, or RR @1y HR 1.32, 95% CI 0.78–2.23 *p* = 0.31
FRAME-AMI [15]	2023	562	STEMI (47%) and NSTEMI (53%)	FFR-guided CompR vs. angio-guided CompR	FFR ≤ 0.80) vs. stenosis > 50% (staged ~40%)	death, MI, and RR @ median FU of 3.5 years HR 0.43, 95% CI 0.25–0.75 *p* = 0.003
COMPLETE-2	recruiting	5100	STEMI and NSTEMI	physiology-guided CompR vs. angio-guided CompR	RFR ≤ 0.89) or FFR ≤ 0.80 vs. stenosis ≥ 50%	CV death, MI, or IDR

## 6. Summary

Table 1 provides an overview of studies investigating the optimal revascularization strategy in patients with MI and multivessel disease.

In summary, for STEMI, the basic question has been well addressed by the COMPLETE trial: in hemodynamically stable patients with STEMI, complete revascularization should occur within 45 days [8]. For the NSTEMI population, this basic question has not been definitively answered yet, and the ongoing COMPLETE-NSTEMI trial is expected to provide insights in the future.

Regarding the question of optimal timing, it appears that in hemodynamically stable patients, a one-time complete revascularization may not be inferior [25,26]. However, the trials were too heterogeneous and too small, and therefore, the data was not robust enough for a definitive conclusion.

Assuming an advantage for complete revascularization, the third question arises: how should the lesions for complete revascularization be optimally identified—angiographically, functionally, or morphologically? The second was defined in relevant studies using thresholds for FFR or RFR. Published data on this matter are inconclusive as well. While the relatively small FRAME-AMI study indicates an advantage for physiology-guided non-culprit lesion PCI, the larger FLOWER-MI study was neutral (neither an advantage nor a disadvantage) [9,15]. Although the FIRE trial also showed an advantage for physiology-guided non-culprit lesion PCI, it is, strictly speaking, not a study investigating the guidance of complete revascularization [22]. Instead, FIRE explored the basic question, except that complete revascularization was guided by physiology, not angiography. In contrast to FLOWER-MI and FRAME-AMI, the control group in FIRE did not undergo routine PCI of non-culprit lesions after culprit lesion PCI. This third question is likely to be clarified by the large COMPLETE-2 trial. Furthermore, previously presented observational data suggest that the selection of non-culprit lesions for revascularization based on morphological criteria indicating a vulnerable plaque could be advantageous. However, there are currently no relevant data from randomized studies on this matter. Nevertheless, ongoing studies are addressing this issue (see the next section).

## 7. Perspective

Two large prospective, multi-center, randomized controlled trials are currently investigating the optimal revascularization strategy for patients with NSTEMI and multivessel disease. The COMPLETE-2 trial (ClinicalTrials.gov: NCT05701358) aims to enroll 5100 patients presenting with STEMI or type 1 NSTEMI within 72 h following a successful culprit-lesion PCI, having at least one additional non-infarct-related coronary artery stenosis >50% in 140 trial sites in 25 countries. Patients will be randomized in a 1:1 ratio into two groups: physiology-guided non-culprit-lesion PCI or angiography-guided PCI. In the physiology-guided group, non-culprit-lesion PCI will be performed if the RFR is ≤0.89 or the FFR is ≤0.80. Patients in the angiography-guided group will undergo routine staged PCI of all qualifying non-culprit-lesions identified before randomization. The primary efficacy endpoint is the time to first occurrence of the composite of cardiovascular death, new myocardial infarction, or ischemia-driven revascularization.

The COMPLETE-NSTEMI trial plans to enroll 3390 NSTEMI patients with multivessel disease having an identifiable culprit lesion across approximately 60 study centers in Germany (ClinicalTrials.gov: NCT05786131). The patients will also be randomized into two groups in a 1:1 ratio. Patients randomized to the complete revascularization group will receive complete revascularization through PCI of all angiographically significant non-culprit lesions (≥70% stenosis in a single view or ≥50% stenosis in two views in a vessel at least 2.5 mm in diameter). This may occur during the index procedure, the index hospitalization, or staged within 45 days after PCI of the culprit lesion. Patients randomized to the culprit lesion-only revascularization group will receive optimal medical therapy without further revascularization of non-culprit lesions. Revascularization of non-culprit lesions will only be permitted if at least one of the following bailout criteria is met: (a) rehospitalization for non-fatal myocardial infarction, (b) rehospitalization for heart failure, or (c) persistent intractable angina pectoris (Canadian Cardiovascular Society grade ≥ III) and evidence of ischemia on non-invasive or invasive ischemia testing. The primary efficacy endpoint is the time to the first occurrence of the composite of cardiovascular death or rehospitalization for non-fatal myocardial infarction.

The two major design differences between both trials are as follows: (a) COMPLETE-2 includes both NSTEMI and STEMI patients, while COMPLETE-NSTEMI only includes NSTEMI patients, and (b) the comparator group for complete revascularization in COMPLETE-2 is a physiology-guided PCI of the non-culprit lesions, while in COMPLETE-NSTEMI, there is no planned PCI of non-culprit lesions.

**Figure 3:**
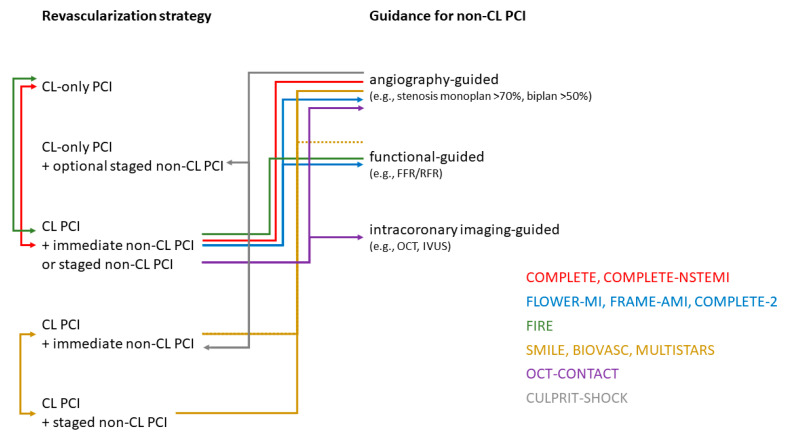
This subway map-like illustration aims to represent the complexity and heterogeneity of randomized controlled trials (RCT) investigating the optimal revascularization strategy for non-culprit lesion percutaneous coronary interventions (PCIs) in patients with myocardial infarction and multivessel disease. The arrows point to the comparator of the RCTs. The arrowless connection signifies the reference to the revascularization strategy or the guidance for non-culprit lesion PCIs. The color coding represents different groups of RCTs. To avoid overwhelming complexity, the depiction excludes the representation of the study population (STEMI, NSTEMI, STEMI and NSTEMI, cardiogenic shock, etc.). However, this difference significantly complicates the interpretation of the studies. Abbreviations: CL = culprit lesion; CMR = cardiac magnetic resonance; PCI = percutaneous coronary intervention; PET = positron emission tomography; SPECT = single-photon emission computed tomography.

Further interesting studies explore the guidance of complete revascularization using intravascular imaging [33]. OCT-CONTACT (ClinicalTrials.gov: NCT04878133) compares immediate OCT-guided complete revascularization with unguided complete revascularization in 460 STEMI patients. FRAME-AMI 2 (ClinicalTrials.gov: NCT05812963) compares IVUS-guided PCI with FFR-guided PCI in 1400 STEMI patients. VULNERABLE (ClinicalTrials.gov: NCT05599061) will randomize 600 STEMI patients with angiographically immediate (40–69% stenosis) non-culprit lesions without hemodynamic significance (FFR >0.80) but with vulnerable plaque morphology observed in OCT. Patients will be assigned to either PCI for these lesions or to receive optimal medical therapy alone. Moreover, OCT sub-studies are also planned in COMPLETE-NSTEMI and COMPLETE-2. The eagerly anticipated results of all these studies will shed more light on the optimal revascularization strategy for acute MI, considering the pathophysiology discussed earlier. This is urgently needed because the complexity of the revascularization strategy (e.g., complete revascularization: yes or no; timing of complete revascularization: immediate or staged; guidance of complete revascularization: angiographic, functional, or intravascular imaging) is enormous (Figure 3).

## Data Availability

Not applicable.

## Abbreviations

BIOVASC	Percutaneous complete revascularization strategies using sirolimus-eluting biodegradable polymer-coated stents in patients presenting with acute coronary syndrome and multivessel disease
COMPARE-ACUTE	Fractional Flow Reserve-Guided Multivessel Angioplasty in Myocardial Infarction
COMPLETE	Complete vs. Culprit-Only Revascularization Strategies to Treat Multivessel Disease after Early PCI for STEMI
COMPLETE-2	Physiology-guided vs. Angiography-guided Non-culprit Lesion Complete Revascularization for Acute MI & Multivessel Disease
COMPLETE-NSTEMI	Complete Revascularization Versus Culprit Lesion Only PCI in NSTEMI
CvLPRIT	Complete vs. Lesion-only Primary PCI trial
DANAMI-3-PRIMULTI	Complete revascularization vs. treatment of the culprit lesion only in patients with ST-segment elevation myocardial infarction and multivessel disease
FIRE	Functional Assessment in Elderly MI Patients with Multivessel Disease
FLOWER-MI	Flow Evaluation to Guide Revascularization in Multivessel ST-Elevation Myocardial Infarction
FORZA	Fractional Flow Reserve vs. Optical Coherence Tomography to Guide Revascularization of Intermediate Coronary Stenoses
FRAME-AMI	Fractional Flow Reserve vs. Angiography-Guided Strategy for Management of Non-Infarction Related Artery Stenosis in Patients with Acute Myocardial Infarction
MULTISTARS AMI	Multivessel Immediate vs. Staged Revascularization in Acute Myocardial Infarction
OCT-CONTACT	OCT Guided vs. COmplete Pci in patieNts With sT-Segment Elevation myocArdial infarCtion and mulTivessel Disease
PRAMI	Preventive Angioplasty in Acute Myocardial Infarction
SMILE	Impact of Different Treatment in Multivessel Non-ST Elevation Myocardial Infarction Patients: One Stage vs. Multistaged Percutaneous Coronary Intervention
VULNERABLE	Treatment of Functionally Non-significant Vulnerable Plaques in Patients With Multivessel ST-elevation Myocardial Infarction
